# Autism from a Biometric Perspective

**DOI:** 10.3390/ijerph7051984

**Published:** 2010-04-28

**Authors:** Nataliya Kostyuk, Rajendram V. Rajnarayanan, Raphael D. Isokpehi, Hari H. Cohly

**Affiliations:** 1 Center for Bioinformatics and Computational Biology, Department of Biology, Jackson State University, 1400 JR Lynch Street, Box 18540, Jackson, MS 39217, USA; E-Mails: nkostyuk@gmail.com (N.K.); raphael.isokpehi@jsums.edu (R.I.); 2 School of Medicine and Biomedical Sciences, University at Buffalo, State University of New York, Buffalo, NY 14214, USA; E-Mail: rajendra@buffalo.edu

**Keywords:** autism, biometric evaluation, electro-photonic emission, gas discharge visualization (GDV)

## Abstract

**Purpose:**

The aim of this pilot study was to test autistic children, siblings and their parents using a biometric device based on the gas discharge visualization (GDV) technique in order to assess their psycho-emotional and physiological functional state based on the activity of the autonomic nervous system.

**Hypothesis:**

We hypothesize that the biometric assessment based on GDV will enable us: (1) to evaluate some specific features associated with autism spectrum disorder (ASD) as well as to compare autistic children to their siblings and to controls; (2) to analyze the differences in individual values of parents of autistic children *versus* parents of normal children.

**Results:**

Out of total of 48 acupuncture points present on ten fingertips of both hands and associated to organs/organ systems, autistic children differed significantly from controls (p < 0.05) in 36 (images without filter) and 12 (images with filter), siblings differed significantly from controls (p < 0.05) in 12 (images without filter) and seven (images with filter), autistic children differed significantly (p < 0.05) from siblings in eight (images without filter) and one (images with filter), fathers of autistic children differed significantly (p < 0.05) from controls in 14 (images without filter) and three (images with filter) and mothers of autistic children differed significantly (p < 0.05) from controls in five (images without filter) and nine (images with filter) acupuncture points.

**Conclusions:**

All compared groups have shown significant difference on both psycho-emotional (images without filter) and physiological (images with filter) levels. However, the differences between autistic children and controls expressed on psycho-emotional level were the most significant as compared to the other groups. Therefore, the activity of the sympathetic autonomic nervous system is significantly altered in children with autism. The biometric method based on GDV is a promising step in autism research that may lead towards creating a disease profile and identify unique signature/biomarker for autism. Further work should involve more participants in order to augment our findings.

## 1. Introduction

According to the United States Department of Developmental Services, the prevalence of autism spectrum disorders increased 556% from 1991 to 1997 [1]. One in every eight boys is diagnosed with autism, and boys are four times more likely to be affected by this disease than girls. Also, one out of every sixty-eight families has a child with autism. Incidences of autism are increasing by 3.8% per year worldwide and by 15% in the USA [1]. The common signs of autism are marked by: (1) qualitative impairment in social interaction; (2) qualitative impairments in communication; (3) restricted repetitive and stereotyped patterns of behavior. Autism covers a continuum of disorders beginning from mild autism and Asperger’s Syndrome to severe autism. Autism Spectrum Disorder is defined only behaviorally, which often contributes to the heterogeneity of cohort studies. Factors such as age, gender, IQ, and behavioral traits often diverge considerably, with non-uniform matching of controls.

**Figure 1.**
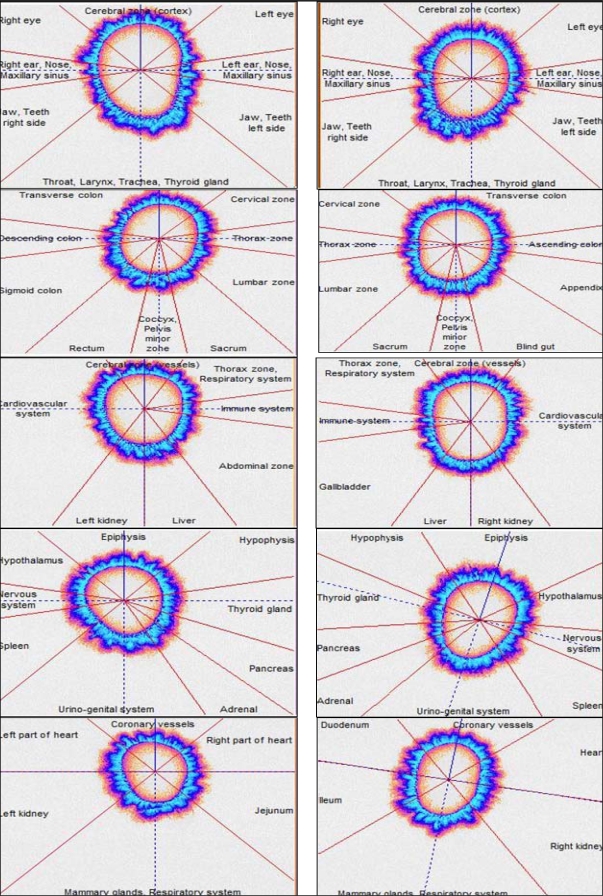
Division of images into sectors after Korotkov K.G.

The six autistic participants, their parents, siblings and control groups were tested using biometric device based on gas discharge visualization (GDV). It is a non-invasive imaging technique used to assess the functional state of human body by recording the responses of the autonomic nervous system to a high intensity electromagnetic field using fingertips. The method is based on acupuncture approach, where organs and organ systems are connected by electron communication. The classification of acupuncture points for GDV has been developed by Mandel and Korotkov and is presented in Figure 1. The respective sectors of the fingertips correspond to specific organs/organ systems including the immune, respiratory, digestive, cardiovascular, central nervous, peripheral nervous, urino-genital, and endocrine systems [2–6].

The aim of our pilot study was to compare autistic individuals with controls and non-autistic siblings as well as to compare their parents with parents who do not have autistic children and analyze statistically the obtained results for possible common difference regarding particular acupuncture regions present on the fingertips.

## 2. Experimental Section

### A. The Institutional Review Board (IRB) Approval

The IRB approval of the consent form was obtained according to the guidelines prescribed by the Review Board at Jackson State University. All participants were residents of Mississippi. The participants of the study and their parents signed a consent form where they agreed that the results of the study may be used for publication or teaching purposes.

### B. Participants

The autistic children in this study were previously diagnosed with mild autism and/or Asperger’s Syndrome. The screenings of autistic children were done randomly. However, the age of the autistic children fell into a range of five to twelve years old, 9.3 being the mean age. The mean age of the control group was the same. Control group for children category had two males and five females. Control groups for mothers and fathers of autistic children were selected randomly from the database of electro-photonic emission of human fingertips of healthy individuals.

### C. Equipment, Software, and Procedure

The study was conducted using a biometric device based on GDV “GDV Compact”. To reduce the barrier of a new setting for autistic children, parents were asked to participate first. Each participant was asked to place each finger correctly on a glass surface. The images of the electro-photonic emissions of all ten fingertips were taken twice. First, we recorded the response of the sympathetic nervous system, which was measured using the properties of electrical conductance of skin in high intensity electromagnetic field. Second, to assess the physiological state of autistic individuals and their parents, we used thin plastic film to separate skin from direct contact with the glass surface thereby enabling recording of the response of the parasympathetic nervous system.

The image of each fingertip is captured individually as a single snapshot with an optical camera that is placed underneath the glass surface on which the participant puts each finger. Under a high intensity electromagnetic field, the finger emits a burst of electrons and photons and electro-photonic emissions are transformed into video-signals, which are recorded in the form of single snapshots or fingertip images called GDV-grams. The data processor utilizes a specialized software complex that permits the calculation of the system parameters. The software GDV Diagram facilitates the implementation of standardized processing of GDV-images. The process involves capturing and filtering GDV-images, obtaining numerical values, creating graphs and diagrams, and saving data as well as transferring data for additional processing.

### D. Statistical Analysis

SAS software was used for statistical analysis of the obtained data, specifically General Linear Model Procedure including Tukey’s and Duncan’s tests.

## 3. Results

The results of the screening of autistic individuals, their parents and siblings as well as control groups are presented graphically in Table 1, Table 2, and Table 3. Five groups were compared: (1) autistic children and controls (marked with green color) (Table 1), (2) siblings from the families with autistic children and controls (marked with blue color) (Table 2), (3) autistic children and siblings (marked with yellow color) (Table 2), (4) fathers of autistic children and controls (marked with grey color) (Table 3), (5) mothers of autistic children and controls (marked with pink color) (Table 3). The comparison of captures without filter and with filter within mentioned groups was done on the level of individual values assigned to the specific acupuncture regions of fingertips. Out of total of 48 acupuncture points present on ten fingertips of both hands and associated to organs/organ systems, autistic children differed significantly from controls (p < 0.05) in 36 (images without filter) and 12 (images with filter), siblings differed significantly from controls (p < 0.05) in 12 (images without filter) and seven (images with filter), autistic children differed significantly (p < 0.05) from siblings in eight (images without filter) and one (images with filter), fathers of autistic children differed significantly (p < 0.05) from controls in 14 (images without filter) and three (images with filter) and mothers of autistic children differed significantly (p < 0.05) from controls in five (images without filter) and nine (images with filter) acupuncture points.

The statistical analysis of data of autistic children *versus* controls in Table 1. (images without filter) has demonstrated statistically significant difference (p < 0.05) for the acupuncture regions corresponding to right eye, right ear, nose, maxillary sinus, jaw and teeth right side, throat, trachea, larynx, thyroid gland, jaw and teeth left side, left ear, left eye, cerebral cortex, thorax zone, lumbar zone, coccyx, pelvis minor zone, blind gut, appendix, ascending colon, transverse colon, descending colon, mammary glands and respiratory system, immune system, liver, cardiovascular system, cerebral vessels, abdominal zone, hypophysis, thyroid, pancreas, adrenal gland, urino-genital system, spleen, nervous system, epiphysis, duodenum, ileum and jejunum. Analysis of the images with filter pointed out statistically significant difference (p < 0.05) for acupuncture regions corresponding to right ear, nose, maxillary sinus, jaw and teeth right side, throat, larynx, trachea, thyroid gland, left eye, lumbar zone, pancreas, spleen, nervous system, ileum, jejunum and right part of heart.

Also, significantly different (p < 0.05) were integral parameters of autonomic tone (activation coefficient) and area of electro-photonic emission (images without filter) (Table 1).

Comparison of siblings with controls (Table 2) showed statistical significance (p < 0.05) of following variables (images without filter): jaw and teeth left side, thorax zone, lumbar zone, appendix, ascending colon, gallbladder, liver, pancreas, spleen, nervous system, epiphysis and jejunum. Statistical analysis of data obtained from the images with filter indicate significant difference (p < 0.05) for right eye, lumbar zone, coccyx, pelvis minor zone, thyroid gland, hypothalamus, mammary glands and respiratory system and jejunum.

Also, significantly different (p < 0.05) were integral parameters of autonomic tone (activation coefficient) and entropy of electro-photonic emission (images with filter) (Table 2).

Within groups of autistic children and siblings (Table 2) statistically significant (p < 0.05) difference was found for acupuncture regions corresponding to throat, larynx, trachea, thyroid gland, descending colon, liver, left kidney, abdominal zone, hypophysis, urino-genital, mammary glands and respiratory system (images without filter) and mammary glands and respiratory system (images with filter).

Also, significantly different (p < 0.05) were integral parameters of autonomic tone (activation coefficient) and area of electro-photonic emission (images without filter) (Table 2).

Fathers of autistic children *versus* control group (Table 3) exhibited statistically significant difference (p < 0.05) in the regions of left ear, nose, maxillary sinus, left eye, thorax zone, sacrum, coccyx, pelvis minor zone, blind gut, appendix, immune system, right kidney, thyroid gland, hypothalamus, epiphysis, mammary glands and respiratory system and heart (images without filter) and throat, larynx, trachea, thyroid gland, mammary glands and respiratory system and heart (images with filter).

Mothers of autistic children *versus* control group (Table 3) demonstrated statistically significant difference (p < 0.05) in acupuncture regions corresponding to right eye, right ear, nose, maxillary sinus, throat, larynx, trachea, thyroid gland, left eye and epiphysis (images without filter) and right eye, jaw and teeth left side, left ear, nose, maxillary sinus, left eye, cervical zone, thorax zone, abdominal zone, pancreas and right part of heart (images with filter).

## 4. Discussion

The GDV biometric technique has been successfully used in psychology and cognitive studies mainly to assess the psycho-emotional and physiological state of an individual as well as to evaluate the changes that take place in a human organism over a period of time. Based on integral parameters such as form and size of electro-photonic emission, symmetry and relationship of the captured image with the rest of the GDV-grams of all fingertips, the presence or absence of aggressive signs and defects with the organs/organ systems one can conclude about the functional state of an individual at the moment of study [7,8].

Autism is a severe neurodevelopmental disorder with the development prior to 3 years of age [9]. The cause of autism remains unknown, and it is a heterogeneous disorder as to its etiology and phenotype. Autistic children are vulnerable to oxidative stress and are easily influenced by genetic, environmental, and immunological factors [10]. Immune [10–13], autoimmune, and infectious factors [14,15] have also been mentioned as playing role in the manifestation of autism. GDV assessment of psycho- emotional and physiological state of autistic individuals confirms that acupuncture regions on fingertips corresponding to the regions of “spleen” and “immune system” significantly differ from the same regions in controls (Table 1). The genetic nature of autism is unclear; there is no single gene that has been found to be associated with autism. Instead, multiple genes have been reported as being associated with autism [16]. Patients with autism can differ in the severity and scope of their symptoms suggesting that multiple factors contribute to explaining the disorder’s symptoms. Environmental aspects, such as mercury, lead, measles, rubella virus, retinoic acid, maternal thalidomide, valproic acid and alcohol use during pregnancy and stress have been implicated in autism [17,18]. In addition, patients with autism are described as having behavior impairments, gastrointestinal deviations [19] and epilepsy [20]. According to GDV testing, autistic children have shown significant difference from normal children in acupuncture regions corresponding to descending, transverse, ascending colon, blind gut, appendix, pelvis minor zone as well as nervous system which may relate to behavioral impairments in autistic individuals. Recent clinical evidence emphasizes the significance of oxidative stress in the development and expression of autism [21]. Interestingly, autistic children in our experimental setting have acupuncture regions corresponding to respiratory system, trachea, larynx, maxillary sinus significantly different from controls and many impaired manifestations in autism relate to weakness of respiratory muscle in autistic patients [22]. There are numerous studies in the medical literature [23–30] that confirm cerebral hypoperfusion (decreased blood flow to the brain) in as many as 86% of individuals with autism [23]. Furthermore, this diminished blood flow typically correlates with many core autistic symptoms. Thus, it has been suggested that abnormal areas in the cerebral cortex are related to the cognitive impairments (such as deficits in language, impaired executive function and abnormal responses to sensory stimuli) observed in autistic children. In fact, sometimes the cerebral blood flow actually decreases, and this appears to be mediated, in part, by inappropriate vasoconstriction (narrowing of blood vessels) instead of vasodilation [31]. Cerebral hypoperfusion appears to lead to cerebral hypoxia (impaired oxygen delivery) to the brain in some autistic individuals. The cause of cerebral hypoperfusion in autistic individuals is unknown, but might be due to inflammation. Inflammation around blood vessels can cause the vessel wall to become stiff and inflexible. Vasculitis decreases the ability of the blood vessel to dilate and can lead to diminished blood flow. Other studies confirm the presence of inflammation in the brain of some autistic individuals. Inflammation, generally associated with increased content of water (edema), can increase the space between cells [32], and might increase the amount of fluid present inside cells [33]. Furthermore, the ability of one brain cell to communicate to another cell is reduced in some autistic children when compared to neurotypical children [34]. Thus, there exists a high probability that inflammation present in the brain of some autistic individuals is leading to diminished blood flow, impaired functional connectivity, impaired cognition, and increased fluid inside brain cells. Researchers at the Pennsylvania School of Medicine have shown constricted blood vessels and low blood flow in autistic individuals via biochemical analysis [35]. Our results shown in Table 1 confirm that there is a significant difference between autistic children and normal children (images without filter) for acupuncture region corresponding to cerebral vessels and cerebral cortex which could imply that autistic children may have malfunctioning blood flow in cerebral vessels and cortex.

## 5. Conclusions

All compared groups have shown significant difference on both psycho-emotional (images without filter) and physiological (images with filter) levels. However, the differences between autistic children and controls expressed on psycho-emotional level were the most significant as compared to the other groups. Therefore, the activity of the sympathetic autonomic nervous system is significantly altered in children with autism. The biometric method based on GDV is a promising step in autism research that may lead towards creating a disease profile and identify unique signature/biomarker for autism. Further work should involve more participants in order to augment our findings.

## Figures and Tables

**Table 1.** Statistically significant difference (p < 0.05) of organs within the groups of autistic children *vs.* control.

**Autistic children*vs.*Controls**						
**Organs/Organ systems**	**F0**	**F1**	**Organs/Organ systems**	**F0**	**F1**
**Right eye**			**Cardiovascular system**		
**Right ear, Nose, Maxillary sinus**			**Cerebral zone (vessels)**		
**Jaw, Teeth right side**			**Abdominal zone**		
**Throat, Larynx, Trachea, Thyroid gland**			**Hypophysis**		
**Jaw, Teeth left side**			**Thyroid gland**		
**Left ear, Nose, Maxillary sinus**			**Pancreas**		
**Left eye**			**Adrenal**		
**Cerebral zone (cortex)**			**Urino-genital system**		
**Thorax zone**			**Spleen**		
**Lumbar zone**			**Nervous system**		
**Coccyx, Pelvis minor zone**			**Epiphysis**		
**Blind gut**			**Duodenum**		
**Appendix**			**Ileum**		
**Ascending colon**			**Mammary glands, Respiratory system**		
**Transverse colon**			**Jejunum**		
**Descending colon**			**Right part of heart**		
**Thorax zone, Respiratory system**			**Activation coefficient**		
**Immune system**			**Integral area**		
**Gallbladder**			**RMS of Integral area**		
**Liver**					

* F0 and F1 are measurement without and with the filter. Green highlights indicate P < 0.05.

**Table 2.** Statistically significant difference (p < 0.05) of organs within groups of siblings *vs*. normal children and autistic children *vs.* siblings.

**Organs/Organ systems**	**Siblings*vs.*control group**		**Autistic group*vs.*siblings**	
	**F0**	**F1**	**F0**	**F1**
**Right eye**				
**Throat, Larynx, Trachea, Thyroid gland**				
**Jaw, Teeth left side**				
**Thorax zone**				
**Lumbar zone**				
**Sacrum**				
**Coccyx, Pelvis minor zone**				
**Appendix**				
**Ascending colon**				
**Descending colon**				
**Gallbladder**				
**Liver**				
**Left kidney**				
**Abdominal zone**				
**Hypophysis**				
**Thyroid gland**				
**Pancreas**				
**Urino-genital system**				
**Spleen**				
**Nervous system**				
**Hypothalamus**				
**Epiphysis**				
**Mammary glands, Respiratory system**				
**Jejunum**				
**Activation coefficient**				
**Integral area**				
**Integral entropy**				

* F0 and F1 are measurements with and without the filter. Cyan and yellow highlights indicate P < 0.05.

**Table 3.** Statistically significant difference (p < 0.05) of organs within groups of fathers *vs.* control and mothers *vs*. control.

**Organs/Organ systems**	**Fathers*vs.*fathers control**		**Mothers*vs.*mothers control**	
	**F0**	**F1**	**F0**	**F1**
**Right eye**				
**Right ear, Nose, Maxillary sinus**				
**Throat, Larynx, Trachea, Thyroid gland**				
**Jaw, Teeth left side**				
**Left ear, Nose, Maxillary sinus**				
**Left eye**				
**Cervical zone**				
**Thorax zone**				
**Sacrum**				
**Coccyx, Pelvis minor zone**				
**Blind gut**				
**Appendix**				
**Immune system**				
**Right kidney**				
**Abdominal zone**				
**Thyroid gland**				
**Pancreas**				
**Hypothalamus**				
**Epiphysis**				
**Mammary glands, Respiratory system**				
**Heart**				
**Right part of heart**				

* F0 and F1 are measurements without and with the filter. Grey and Orange highlights indicate P < 0.05.

## References

[1] Stokstad E Development. New hints into the biological basis of autism Science 2001 294 34 37 11588233 10.1126/science.294.5540.34

[2] Bell IR Lewis DA Brooks AJ Schwartz GE Gas discharge visualization evaluation of ultramolecular doses of homeopathic medicines under blinded, controlled conditions J. Altern. Complement. Med 2003 9 25 38 12676033 10.1089/107555303321222928

[3] Dobson P O’Keeffe E The efficacy of the gas discharge visualisation technique as a measure of physical and mental health Proceedings of 18th IEEE Symposium on Computer Based Medical Systems IEEE Computer Society Dublin, Ireland23–24 June, 2005455 457

[4] Korotkov KG Williams B Wisnesky LA Assessing biophysical energy transfer mechanisms in living systems: the basis of life processes J. Altern. Complement. Med 2004 10 49 57 15025878 10.1089/107555304322848959

[5] Korotkov KG Human Energy Field: Study with GDV Bioelectrography Backbone Publishing Co. Fair Lawn, NJ, USA 2002

[6] Korotkov KG Popechitelev EP Method for gas-discharge visualization and automation of the system of realizing it in clinical practice Med. Tekh 2002 1 21 25 11898657

[7] Ivanov OC Yusubov RR Akhmetelli GG Diagnostics of psycho-physiological state of a human on the basis of GDV diagnostics Proceedings of XII International Scientific Congress on Bio-electrography Saint Petersburg, Russia July 3–4, 2008 40 42

[8] Ivanov OC Yusubov RR Akhmetelli GG Interpretation of psycho-emotional state of a human on the basis of GDV diagnostics Proceedings of XII International Scientific Congress on Bio-electrography Saint Petersburg, Russia July 3–4, 2008 43 46

[9] Lord C Cook EH Leventhal BL Amaral DG Autism spectrum disorders Neuron 2000 28 355 363 11144346 10.1016/s0896-6273(00)00115-x

[10] Cohly HH Panja A Immunological findings in autism Int. Rev. Neurobiol 2005 71 317 341 16512356 10.1016/s0074-7742(05)71013-8

[11] Keller F Persico AM The neurobiological context of autism Mol. Neurobiol 2003 28 1 22 14514983 10.1385/MN:28:1:1

[12] Korvatska E Van de WJ Anders TF Gershwin ME Genetic and immunologic considerations in autism Neurobiol. Dis 2002 9 107 125 11895365 10.1006/nbdi.2002.0479

[13] Krause I He XS Gershwin ME Shoenfeld Y Brief report: immune factors in autism: a critical review J. Autism. Dev. Disord 2002 32 337 345 12199139 10.1023/a:1016391121003

[14] Wakefield AJ Montgomery SM Autism, viral infection and measles-mumps-rubella vaccination Isr. Med. Assoc. J 1999 1 183 187 10731332

[15] Fombonne E Are measles infections or measles immunizations linked to autism? J. Autism. Dev. Disord 1999 29 349 350 10478736 10.1023/a:1022123822135

[16] Lamb JA Moore J Bailey A Monaco AP Autism: recent molecular genetic advances Hum. Mol. Genet 2000 9 861 868 10767308 10.1093/hmg/9.6.861

[17] London EA The environment as an etiologic factor in autism: A new direction for research Environ. Health Perspect 2000 108 401 404 10852835 10.1289/ehp.00108s3401 PMC1637814

[18] Mutter J Naumann J Schneider R Walach H Haley B Mercury and autism: accelerating evidence? Neuro. Endocrinol. Lett 2005 26 439 446 16264412

[19] White JF Intestinal pathophysiology in autism Exp. Biol. Med. (Maywood) 2003 228 639 649 12773694 10.1177/153537020322800601

[20] Tuchman R Rapin I Epilepsy in autism Lancet. Neurol 2002 1 352 358 12849396 10.1016/s1474-4422(02)00160-6

[21] McGinnis WR Oxidative stress in autism Altern. Ther. Health Med 2004 10 22 36 15624347

[22] Constipation, Behavior, Sleep, Autism and Breathing Available online: http://advancecentres.blogspot.com/2009/06/constipation-behaviour-sleep-autism-and.htm (accessed on 12 December 2009).

[23] Zilbovicius M Boddaert N Belin P Poline JB Remy P Mangin JF Thivard L Barthélémy C Samson Y Temporal lobe dysfunction in childhood autism: a PET study. Positron emission tomography Am. J. Psychiatry 2000 157 1988 1993 11097965 10.1176/appi.ajp.157.12.1988

[24] Wilcox J Tsuang MT Ledger E Algeo J Schnurr T Brain perfusion in autism varies with age Neuropsychobiology 2002 46 13 16 12207141 10.1159/000063570

[25] Starkstein SE Vazquez S Vrancic D Nanclares V Manes F Piven J Plebst C SPECT findings in mentally retarded autistic individuals J. Neuropsychiatry Clin. Neurosci 2000 12 370 375 10956571 10.1176/jnp.12.3.370

[26] Ohnishi T Matsuda H Hashimoto T Kunihiro T Nishikawa M Uema T Sasaki M Abnormal regional cerebral blood flow in childhood autism Brain 2000 123 1838 1844 10960047 10.1093/brain/123.9.1838

[27] Critchley HD Daly EM Bullmore ET Williams SC van Amelsvoort T Robertson DM Rowe A Phillips M McAlonan G Howlin P Murphy DG The functional neuroanatomy of social behaviour: changes in cerebral blood flow when people with autistic disorder process facial expressions Brain 2000 123 2203 2212 11050021 10.1093/brain/123.11.2203

[28] Pierce K Haist F Sedaghat F Courchesne E The brain response to personally familiar faces in autism: findings of fusiform activity and beyond Brain 2004 127 2703 2716 15319275 10.1093/brain/awh289

[29] Boddaert N Zilbovicius M Functional neuroimaging and childhood autism Pediatr. Radiol 2002 32 1 7 11819054 10.1007/s00247-001-0570-x

[30] Hashimoto T Sasaki M Fukumizu M Hanaoka S Sugai K Matsuda H Single-photon emission computed tomography of the brain in autism: effect of the developmental level Pediatr. Neurol 2000 23 416 420 11118797 10.1016/s0887-8994(00)00224-1

[31] Weizman A Weizman R Szekely GA Wijsenbeek J Livni E Abnormal immune response to brain tissue antigen in the syndrome of autism Am. J. Psychiatry 1982 139 1462 1465 6182806 10.1176/ajp.139.11.1462

[32] Lu G Qian X Berezin I Telford GL Huizinga JD Sarna SK Inflammation modulates *in vitro* colonic myoelectric and contractile activity and interstitial cells of Cajal Am. J. Physiol 1997 273 G1233 G1245 9435548 10.1152/ajpgi.1997.273.6.G1233

[33] Hendry J DeVito T Gelman N Densmore M Rajakumar N Pavlosky W Williamson PC Thompson PM Drost DJ Nicolson R White matter abnormalities in autism detected through transverse relaxation time imaging Neuroimage 2006 29 1049 1057 16214373 10.1016/j.neuroimage.2005.08.039

[34] Just MA Cherkassky VL Keller TA Minshew NJ Cortical activation and synchronization during sentence comprehension in high-functioning autism: evidence of underconnectivity Brain 2004 127 1811 1821 15215213 10.1093/brain/awh199

[35] Penn Researchers Find Link between Autism and Abnormal Blood-Vessel Function and Oxidative StressAvailable online: http://www.uphs.upenn.edu/news/News_Releases/aug06/autbldvsl.htm (accessed on 12 December 2009).

